# Anti-cancer activities of Brassica juncea leaves in vitro

**DOI:** 10.17179/excli2016-586

**Published:** 2016-11-15

**Authors:** Youngeun Kwak, Jungjae Lee, Jihyeung Ju

**Affiliations:** 1 Department of Food and Nutrition, Chungbuk National University, 1 Chungdae-Ro, Seowon-Gu, Cheongju, 28644, Korea

**Keywords:** mustard leaf, human cancer cell, growth, apoptosis, angiogenesis, metastasis

## Abstract

Mustard (*Brassica juncea*) leaves are commonly consumed in different Asian and African countries. Cancer is a major burden of disease worldwide, and the colorectal and lung cancers are the leading cause of morbidity and mortality among cancers. In the current study, we aimed to investigate the effects of ethanol extract of mustard leaf (MLE) on the growth, angiogenic, and metastatic potentials of HCT116 colorectal carcinoma and H1299 non-small cell lung carcinoma cells *in vitro*. Treatment of HCT116 and H1299 cells with MLE inhibited cell growth in a dose-dependent manner (in the range of 175-700 µg/ml, by 39-86 %) and anchorage-independent colonization (at 700 µg/ml, by 56-86 %). Induction of apoptosis by MLE was evidenced by heterogeneous and condensed nucleus morphology, increased 4′,6-diamidino-2-phenylindole dihydrochloride staining intensity, and elevated sub-G1 cell population. In both HCT116 and H1299 cells, treatment with MLE markedly suppressed the secretion of key pro-angiogenic factors, such as vascular endothelial cell growth factor (by >92 %) and basic fibroblast growth factor (by 73-94 %). MLE was also effective in inhibiting critical events during metastasis, such as invasion (by 18-33 % in HCT116 and H1299), migration (45-82 % in H1299), and adhesion (by 17-45 % in HCT116 and H1299). These results indicate that MLE possesses *in vitro *anti-cancer activities against colon and lung cancers. It needs to be verified whether similar effects are reproduced *in vivo*.

## Introduction

Cancer is a major burden of disease worldwide, and the colorectal and lung cancers are the leading cause of morbidity and mortality among cancers (Jemal et al., 2009[15]). Carcinogenesis is recognized as a multistep process where normal cells evolve progressively to a tumorigenic and ultimately malignant state by sustaining unrestricted growth, resisting apoptosis, inducing angiogenesis, and activating metastasis (Hanahan and Weinberg, 2011[11]). Unrestricted growth is arguably the most fundamental trait of neoplastic cells, which is partially acquired by resisting a programmed cell death, called apoptosis (Hanahan and Weinberg, 2011[11]). Angiogenesis is a process where new blood vessels are formed from existing ones within primary tumor and plays a key role in expanding the neoplastic cell growth by supplying oxygen and nutrients and in mediating metastatic spread of neoplastic cells (Hanahan and Weinberg, 2011[11]; Folkman, 2002[6]). Metastasis is a complex phenomenon where neoplastic cells grown in a primary tumor invade surrounding tissues, penetrate blood vessels, exit vessels at distant sites, and form secondary tumors (Hanahan and Weinberg, 2011[11]; Kumar and Weaver, 2009[21]). Diet is regarded as an important modifying factor in the etiology of cancers (McCullough and Giovannucci, 2004[27]). Identification of effective and safe dietary compounds targeting distinct properties of cancer cells acquired during the multistep carcinogenesis would have significant implication in cancer prevention and treatment.

Mustard *(Brassica juncea) *leaves, one of the cruciferous vegetables, are consumed in different Asian and African countries (Tiku et al., 2008[33]; Lee et al., 2010[23]; Yokozawa et al., 2003[38]; Grubben and Denton, 2004[9]). High intake of cruciferous vegetables has been inversely associated with the risk of several cancers, most consistently colon and lung cancers (Higdon et al., 2007[12]). Mustard leaf extract (MLE) has been reported to possess different biological activities, including antioxidant (Kim et al., 2003[19]; Yokozawa et al., 2002[37], 2003[38]; Tiku et al., 2008[33]), anti-inflammatory (Kim et al., 2005[18]), renal ischemia-protective (Yokozawa et al., 2002[36]), and anti-depressant activities (Thakur et al., 2014[32]). Cytotoxic effects of MLE have also been reported in different cancer cells, including colon, lung, gastric, and breast cancer cells (Kim et al., 2007[17]). However, anti-cancer effects of MLE other than the cytotoxicity have not been reported. 

The objective of the current study was to systematically evaluate the effects of MLE on the growth, anchorage-independent colony formation, apoptosis, pro-angiogenic factor secretion, invasion, migration, and adhesion of human colon and lung cancer cells *in vitro*.

## Materials and Methods

### Preparation of mustard leaf extract

Green mustard leaves (purchased from a local market) were washed, freeze-dried (PH1316, IshinBioBase, Yangju, Korea), and extracted with 70 % ethanol at room temperature for 4 h. After centrifugation at 3,000⨯*g* for 3 min, the supernatant was dried using a speed vacuum (NB-503CIR, N-bioteck, Bucheon, Korea) to remove the ethanol. The resultant extract was weighed (34.7 ± 0.5 % of extraction yield calculated by the mass of extract/the mass of dried leaves) and kept at -70 °C for further analysis. 

### Cell culture and general scheme of treatment

HCT116 human colorectal carcinoma cells and H1299 human non-small cell lung carcinoma cells (Korean Cell Line Bank, Seoul, Korea) were grown in Macoy's 5A (for HCT116) and RPMI (for H1299) medium (Gibco Co., Rockville, MD, USA) containing 10 % fetal bovine serum (FBS; Thermo Scientific, Logan, UT, US) and 1 % penicillin/streptomycin (Welgene Inc., Daegu, Korea) at 37 °C in a humidified 5 % CO_2_. The dried extracts were reconstituted in dimethyl sulfoxide (DMSO) not exceeding the final DMSO concentration of 0.2 % (v/v). The effects of MLE on pro-angiogenic factor secretion, invasion, migration, and adhesion were studied at a maximum of 24 h time point when HCT116 and H1299 cell viability was not significantly affected by MLE at the concentration range used (175-700 µg/ml).

### Cell viability assay

To determine the effect of MLE on anchorage-dependent cell growth, the 3-[4,5-dimethylthiazol-2-yl]-2,5-diphenyl tetrazolium bromide (MTT) assay was performed as previously described (Ju et al., 2012[16]). Briefly, HCT116 and H1299 cells were plated in 96-well plates (5 × 10^3^ cells/well) and allowed to attach for 24 h. The culture media were replaced with fresh serum free media containing different concentrations of MLE (0, 175, 350, and 700 µg/ml). Cells were then incubated with a respective concentration of MLE for 72 and 96 h. The media was removed, and 0.5 mg/mL of MTT-containing fresh media was added in each well. After 4 h incubation at 37 °C, the water insoluble formazan product was produced by metabolically active cells and dissolved with the addition of DMSO. Viable cells were quantified spectrophotometically using a plate reader (Bio-Rad Laboratories, Hercules, CA, US) at the wavelength of 540 nm.

### Soft agar colony formation assay

To determine the effect of MLE on anchorage-independent cell growth, a soft agar assay was performed as previously described (Irons et al., 2010[14]; Kwak and Ju, 2015[22]). Briefly, culture media containing 0.6 % agar (DC Chemical Co., Seoul, Korea) was pre-solidified as a base layer in 6-cm culture dishes. HCT116 (6 × 10^4^ cells/well) and H1299 (8 × 10^3^ cells/well) cells were then suspended in culture media containing 0.3 % agar and layered onto the base layer. Serum complete media containing MLE at the concentration of 700 µg/ml was applied on top of the 0.3 % agar layer, and then cells were cultured for 21 days. Colonies were stained using 0.05 % crystal violet in 20 % methanol for 1 h, and the images of 4 random fields per well were captured (iSolution Lite software, IMT i-solution, Burnarby, Canada). The number and size (in the largest diameter) of colonies were quantified using phase contrast time-lapse microscopy (X100; Primo Vert, Carl Zeiss, Oberkochen, Germany). 

### Staining of cell nucleus 

To determine the effect of MLE on apoptosis, 4′,6-diamidino-2-phenylindole dihydrochloride (DAPI) staining assay was performed as described previously (Toton et al., 2013[34]). Briefly, HCT116 and H1299 cells on 8-well chamber slide (7 × 10^3^ cells/well) were incubated with serum free media containing different concentrations of MLE (0, 175 and 700 µg/ml) for 24 h. Apoptotic cells were visualized by staining the double-stranded DNA of cells with impaired membranes using a fluorescent dye, DAPI (Sigma-Aldrich). Then, cells were washed with PBS, fixed with 4 % formaldehyde (Sigma-Aldrich) for 15 min at room temperature, and incubated with DAPI. Cells with degraded or condensed nuclei (Fesik, 2005[4]; Florent et al., 1999[5]) were observed under confocal laser scanning microscopy (X200; MRC-1024, Bio-Rad Laboratories), and the staining intensity of DAPI was quantified using Image-J software (NIH, Bethesda, MD, USA). 

### Cell cycle analysis

To determine the effect of MLE on cell cycle, starved HCT116 and H1299 cells (3 × 10^5^ cells/well) were cultured in serum complete media containing 0 or 700 µg/ml of MLE for 72 h (HCT116) or 48 h (H1299). Cells were then harvested by trypsinization, fixed in 70 % ethanol overnight at -20 °C, incubated with Ribonuclease A (1 µg/ml; Sigma-Aldrich) and propidium iodine (50 µg/ml; Sigma-Aldrich) for 20 min in the dark at room temperature. Cell population at Sub-G1, G1/G0, S, and G2/M phases were identified according to the DNA content using a flow cytometer (FACS Calibur-S System, BD Biosciences, Heidelberg, Germany) and Cell Quest Pro software (BD Biosciences).

### Enzyme linked immunosorbent assay for measurement of pro-angiogenic factor levels

To determine the effect of MLE on the secretion of key pro-angiogenic factors, such as vascular endothelial cell growth factor (VEGF) and basic fibroblast growth factor (bFGF) (Folkman, 2002[6]), enzyme linked immunosorbent assay (ELISA) was performed. Briefly, HCT116 and H1299 cells were plated in 24-well plates (5 × 10^4^ cells/well) and incubated with serum free media containing different concentrations of MLE (0-700 µg/ml) for 24 h. The media was then collected and centrifuged at 3,000⨯g for 1 min. Levels of VEGF and bFGF were measured using ELISA kits (Koma Biotech Inc., Seoul, Korea) according to the manufacturer's instruction. 

### Transwell invasion assay

To determine the effect of MLE on cell invasion, a transwell assay was performed, as previously described (Ju et al., 2012[16]). Briefly, transwell chambers (8 µm pore size; Corning Inc.) were coated with matrigel (1 mg/ml, Sigma-Aldrich) for 2 h at 37 °C. HCT116 and H1299 cells (1 × 10^6^ cells/well) were loaded in the matrigel-coated inner chambers containing serum free media with MLE (0-700 µg/ml). Cells were then allowed to invade through the matrigel layer toward the outer chamber containing serum complete media. At 24 h after the treatment, the cells still present on the upper surface of the inner chamber were removed using cotton swabs. Then, the cells penetrated to the lower surface of inner chamber and are further present in the outer chamber were stained with 0.2 % crystal violet in 20 % methanol for 1 h. After washing the stained cells with PBS, the dye was released from the cells by the addition of 1 % sodium dodecyl sulfate (SDS; Sigma-Aldrich). Invaded cells were quantified spectrophotometically using a plate reader (Bio-Rad Laboratories) at the wavelength of 540 nm. 

### Wound healing cell migration assay

To determine the effect of MLE on cell migration, a wound healing assay was performed as described previously (Kwak and Ju, 2015[22]). Briefly, HCT116 and H1299 cells on 6-well plates (5 × 10^3^ cells/well) were scratched using a yellow pipette tip in order to introduce a wound. Detached floating cells were removed by washing with PBS. The attached cells were then maintained with serum free media containing different concentrations of MLE (0-700 µg/ml) and permitted to migrate for 24 h. The images of wound were captured by iSolution Lite software (IMT i-solution, Burnarby, Canada), and the wound closure for 24 h was estimated using Image-J software (NIH). 

### Cell adhesion assay

To determine the effect of MLE on cell adhesion, an assay using fibronectin-coated plate was performed as previously described (Matsuura et al., 2006[26]; Kwak and Ju, 2015[22]). Briefly, 1 μg/ml fibronectin (Sigma-Aldrich) and 0.5 % bovine serum albumin (Sigma-Aldrich) were sequentially added in 96-well plates with 2 h interval at 37°C. HCT116 and H1299 cells were mixed with serum complete media containing MLE (0-700 µg/ml) and seeded in the fibronectin-coated 96-well plates (1 × 10^3^ cells/well). After 2 h, unattached cells were washed out with PBS. Attached cells were incubated with 0.2 % crystal violet (in 20 % methanol) for 10 min at room temperature. After dissolving the stained cells by 1 % SDS (Sigma-Aldrich), adherent cells were quantified spectrophotometically using a plate reader (Bio-Rad Laboratories) at the wavelength of 540 nm. 

### Statistical analyses

Data were present as mean ± SEM of at least three determinations. Two groups were compared by Student's t-test, and multiple groups were compared by one-way ANOVA followed by Duncan test. The relationship between a dose and response was assessed by regression analysis. Significance was achieved at p values less than 0.05.

## Results

### MLE inhibited anchorage-dependent and -independent growth in human colon and lung cancer cells 

**Figure 1:**
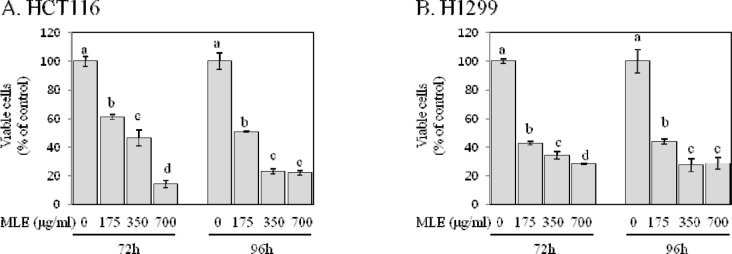
Effect of MLE on the anchorage-dependent growth of HCT116 human colon cancer cells and H1299 human lung cancer cells. HCT116 (A) and H1299 (B) cells were incubated with different concentrations of MLE (0-700 µg/ml) for 72 h and 96 h. Viable cells were determined by the MTT assay as described in Materials and Methods. Statistically significant differences among groups were indicated with different letters (a-d, p<0.05 by Duncan test).

Cancer cells have an ability to grow anchorage-dependently and -independently (Hanahan and Weinberg, 2011[11]). We evaluated the inhibitory effect of MLE against the anchorage-dependent and -independent growth by a typical MTT and soft agar colony formation assay, respectively. In HCT116 human colon cancer cells, the treatment with MLE at the concentration of 175, 350, and 700 µg/ml for 72 h and 96 h significantly inhibited the anchorage-dependent growth by 39-86 %, showing estimated IC_50_ values of 253 µg/ml at the 72 h time point and 153 µg/ml at the 96 h time point (Figure 1(Fig. 1)). MLE was also effective in inhibiting the growth of H1299 human lung cancer cells; the treatment with MLE at the concentration of 175, 350, and 700 µg/ml for 72 h and 96 h inhibited the growth by 56-72 % (IC_50_ value, ~130 µg/ml) (Figure 1(Fig. 1)). In the following regression analysis, a significant dose-response relationship was detected in both HCT116 and H1299 cells (R^2^>0.85, p<0.001).

**Figure 2:**
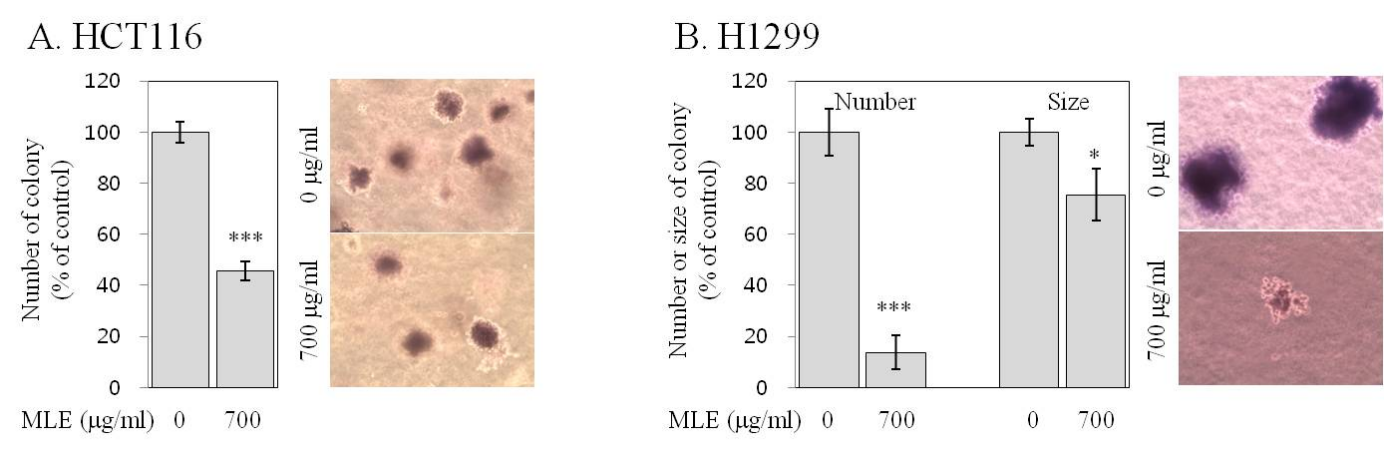
Effect of MLE on the anchorage-independent colony formation of HCT116 human colon cancer cells and H1299 human lung cancer cells. HCT116 (A) and H1299 (B) cells on soft agar were incubated with different concentrations of MLE (0 or 700 µg/ml) for 21 days. The number (in HCT116 and H1299 cells) and size (in H1299 cells) of colonies stained with crystal violet were determined under phase contrast time-lapse microscopy (X100), and representative colonies were present. Statistically significant differences between untreated control and MLE-treated cells were indicated with asterisks (*p<0.05, ***p<0.001, by two-tailed student t-test).

At being cultured for 21 days, both HCT116 and H1299 cells formed a substantial number of colonies in soft agar (Figure 2(Fig. 2)), indicating their property of anchorage-independent growth (Ballet et al., 1981[1]). The treatment with MLE at the concentration of 700 µg/ml significantly reduced the number of colonies by 56 % in HCT116 cells and by 86 % in H1299 cells (Figure 2(Fig. 2)). In H1299 cells producing much bigger size of colonies than HCT116 cells (Kwak and Ju, 2015[22]), the treatment with MLE at the concentration of 700 µg/ml also reduced the size of colonies significantly (by 25 %; Figure 2B(Fig. 2)). 

### MLE induced apoptosis in human colon and lung cancer cells

**Figure 3:**
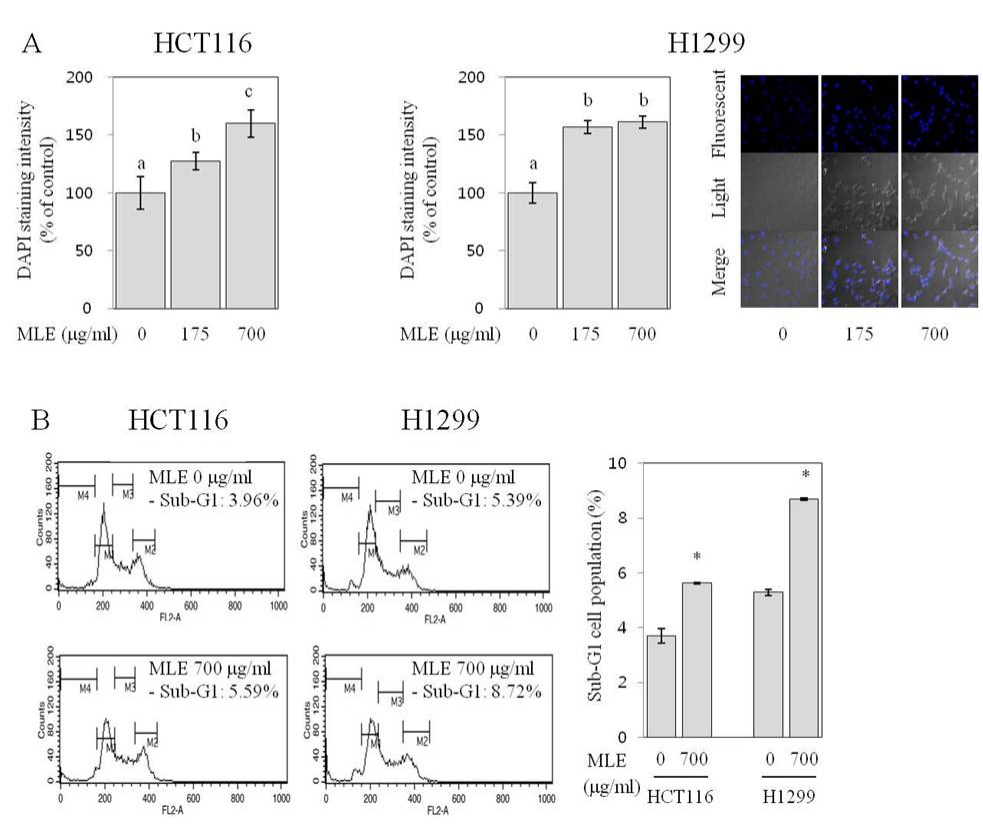
Effect of MLE on apoptosis in HCT116 human colon cancer cells and H1299 human lung cancer cells. DAPI staining intensity (A) of HCT116 and H1299 cells incubated with 0, 175, and 700 µg/ml of MLE for 48 h was estimated, and representative nuclear morphology of H1299 cells was shown on the right panel. Statistically significant differences among groups were indicated with different letters (a-c, p<0.05 by Duncan test). Cell cycle distribution (B) of HCT116 and H1299 cells treated with 0 or 700 µg/ml concentration of MLE was analyzed, and the cell population ( %) at sub-G1 (M4) phase was shown. Statistically significant differences between untreated control and MLE-treated cells were indicated with asterisks (*p<0.05 by two-tailed student t-test).

Loss of apoptotic function is a critical feature of cancer cells and leads to malignant cell growth (Fesik, 2005[4]). We next investigated the apoptosis-inducing activities of MLE by visualizing cell nuclei using a DNA-staining dye, DAPI. The control HCT116 (data not shown) and H1299 cells (Figure 3A(Fig. 3)) presented a typical morphology of healthy cancer cells with homogeneous and symmetric shape of nuclei. The cells treated with MLE at 175 and 700 µg/ml, however, displayed morphological characteristics of apoptotic cells with heterogeneous and condensed nuclear chromatin (Figure 3A(Fig. 3)) (Fesik, 2005[4]; Florent et al., 1999[5]). Apoptotic cells are known to show increased DAPI staining intensity due to their increased membrane permeability (Hotz et al., 1994[13]). The nucleus of control HCT116 and H1299 cells were stained with DAPI very weekly, as expected. On the other hand, the nucleus of cells treated with MLE at the concentrations of 175 and 700 µg/ml showed increased DAPI staining intensity in both HCT116 (to 127-160 % of the control; p<0.05; Figure 3A(Fig. 3)) and H1299 cells (to 157-161 % of the control; p<0.05; Figure 3A(Fig. 3)), indicating the induction of apoptosis by MLE. Cell cycle analyses also revealed the apoptosis-inducing activity of MLE; MLE (at 700 µg/ml) increased the number of cells in the sub-G1 phase (to ~1.5-fold of control HCT116 and H1299 cells, Figure 3B(Fig. 3)). Cell populations at other phases were not significantly changed by the treatment with MLE (data are not shown).

### MLE decreased the secretion of pro-angiogenic factors in human colon and lung cancer cells 

**Figure 4:**
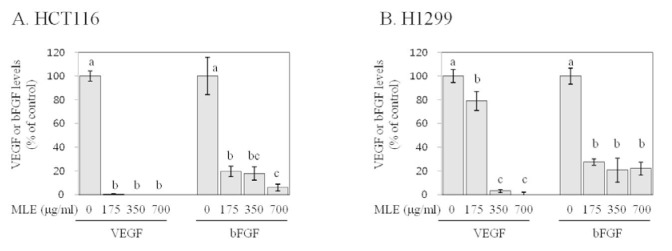
Effect of PLE on pro-angiogenic factor secretion of HCT116 human colon cancer cells and H1299 human lung cancer cells. HCT116 (A) and H1299 (B) cells were incubated with different concentrations of MLE (0-700 µg/ml) for 24 h. The culture media was then collected, and levels of VEGF and bFGF were determined by ELISA. Statistically significant differences among groups were indicated with different letters (a-c, p<0.05 by Duncan test).

Angiogenesis is triggered by the local production of key pro-angiogenic factors from tumor cells, including VEGF and bFGF (Hanahan and Weinberg, 2011[11]). We investigated the effects of MLE on VEGF and bFGF secretion of HCT116 and H1299 cells by ELISA. Both control HCT116 and H1299 cells secreted appreciable levels of VEGF (~40 pg/ml in HCT116 and ~80 pg/ml in H1299) and bFGF (~54 pg/ml in HCT116 and ~67 pg/ml in H1299). Treatment of HCT116 and H1299 cells with MLE at the concentrations of 175, 350, and 700 µg/ml virtually nullified the VEGF secretion (by >92 %, Figure 4(Fig. 4)) and markedly suppressed the bFGF secretion (by 73-94 %, Figure 4(Fig. 4)). 

### MLE inhibited invasion in human colon and lung cancer cells

**Figure 5:**
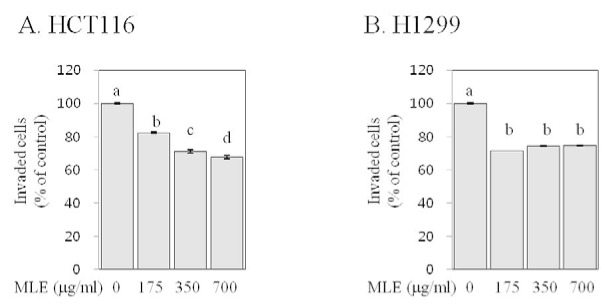
Effect of MLE on invasion in HCT116 human colon cancer cells and H1299 human lung cancer cells. HCT116 (A) and H1299 (B) cells were loaded in the matrigel-coated inner chambers containing serum free media with different concentrations of MLE (0-700 µg/ml) and then allowed to invade through the matrigel layer toward the outer chamber containing serum complete media. After 24 h, cells present in the lower surface of inner chamber and the outer chamber were stained with 0.2 % crystal violet. After dissolving the dye with 1 % SDS, invaded cells were quantified by reading the absorbance at 540 nm using using a plate reader. Statistically significant differences among groups were indicated with different letters (a-d, p<0.05 by Duncan test).

Invasion of cells grown in the primary tumor into surrounding stroma is the first step of cancer metastasis (Hanahan and Weinberg, 2011[11]). We studied the effects of MLE on the invasion of human cancer cells by a transwell assay. Both control HCT116 and H1299 cells displayed invasive properties, as previously reported (Qian et al., 2013[28]; Liao et al., 2015[25]). Treatment with MLE at 175, 350, and 700 µg/ml concentrations suppressed the invasion of HCT116 cells by 18-33 % and H1299 cells by 25-28 % (Figure 5(Fig. 5)).

### MLE inhibited migration in human colon and lung cancer cells

**Figure 6:**
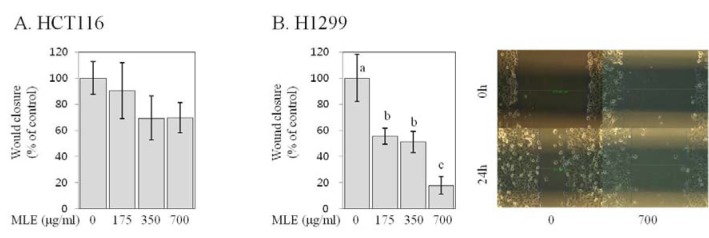
Effect of MLE on migration in HCT116 human colon cancer cells and H1299 human lung cancer cells. HCT116 (A) and H1299 (B) cells were incubated with different concentrations of MLE (0 or 700 µg/ml) for 24 h. The closure of wound for 24 h was estimated, and representative image of wound of H1299 cells (at 0 h and 24 h time points) were shown. Statistically significant differences among groups were indicated with different letters (a-c, p<0.05 by Duncan test).

Migration of cancer cells is prominent event in metastatic cascade (Hanahan and Weinberg, 2011[11]). We evaluated the effects of MLE on the migration of human cancer cells by a wound healing assay. Both control HCT116 and H1299 cells displayed migrative properties, as observed previously (Kwak and Ju, 2015[22]). The treatment of H1299 cells with MLE at the concentrations of 175, 350, and 700 µg/ml significantly reduced the wound closure by 45-82 % (Figure 6(Fig. 6)) in a dose-dependent manner (R^2^>0.8, p<0.001 by regression analysis). In HCT116 cells migrating slowly compared to H1299 cells (Kwak and Ju, 2015[22]), the treatment with MLE only resulted in a trend of reducing the wound closure (at ≥350 µg/ml), but the changes were not statistically significant (Figure 6(Fig. 6)).

### MLE inhibited adhesion in human colon and lung cancer cells 

**Figure 7:**
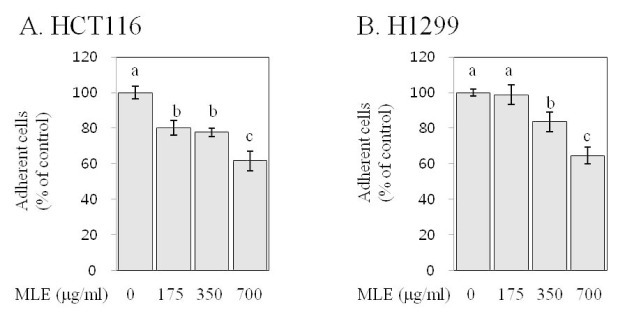
Effect of MLE on adhesion in HCT116 human colon cancer cells and H1299 human lung cancer cells. HCT116 (A) and H1299 (B) cells suspended in serum complete media containing different concentrations of MLE (0-700 µg/ml) were added into plate coated with fibronectin. After 2 h, attached cells on the bottom of plate were quantified. Statistically significant differences among groups were indicated with different letters (a-c, p<0.05 by Duncan test).

Cell adhesion to extracellular matrix is a critical step during metastasis (Hanahan and Weinberg, 2011[11]). We determined the effect of MLE on the adhesion of human cancer cells to fibronectin, a key adhesive glycoprotein of extracellular matrix (Ruoslahti, 1984[29]). The treatment with MLE at 175, 350, and 700 µg/ml concentrations inhibited the adhesion by 20-39 % (p<0.05, Figure 7(Fig. 7)). In H1299 cells, MLE at 350 and 700 µg/ml concentrations was effective in inhibiting the adhesion (by 17-45 %, p<0.05).

## Discussion

Cancer arises from normal cells as a consequence of multistep carcinogenesis (Hanahan and Weinberg, 2011[11]). Critical properties that cells attain sequentially to become tumorigenic and ultimately malignant during the multistep carcinogenesis include sustaining unrestricted growth, resisting apoptosis, inducing angiogenesis, and activating metastasis (Hanahan and Weinberg, 2011[11]). In the present study, we aimed to investigate inhibitory activities of MLE against such critical properties using highly proliferative, angiogenic, and metastatic human cancer cells lines, HCT116 (colon cancer cells) and H1299 (lung cancer cells) (Li et al., 2013[24]; Tang et al., 2012[31]; Xia et al., 2016[35]; Kim et al., 2014[20]).

Uncontrolled growth and inactivated apoptosis are key features of cancer cells (Hanahan and Weinberg, 2011[11]). The inhibition of growth and restoration of apoptosis in cancer cells are, therefore, regarded as an effective strategy for cancer prevention and treatment (Fesik, 2005[4]; Hanahan and Weinberg, 2011[11]). The results of our study indicated that MLE exhibited strong growth-inhibitory (Figures 1(Fig. 1)&2(Fig. 2)) and apoptotic-inducing activities (Figure 3(Fig. 3)) in both HCT116 and H1299 cells. Since the loss of apoptotic function contributes to the uncontrolled growth of cancers (Hanahan and Weinberg, 2011[11]), our findings suggest that the growth-inhibitory activities of MLE may be acquired at least partially by its apoptosis-inducing activity. The anchorage-independent growth *in vitro *is known to be well correlated with tumorigenecity *in vivo *(Ballet et al., 1981[1]). Our findings on the inhibition of anchorage-independent growth of HCT116 and H1299 cells by MLE (Figure 2(Fig. 2)) suggest inhibitory activities of MLE against tumorigenic potentials of colon and lung cancer cells (Fesik, 2005[4]). Although cytotoxic effects of MLE have been reported previously in colon, lung, gastric, and breast cancer cells (Kim et al., 2007[17]), this is the first report, to the best of our knowledge, on the inhibitory activity against anchorage-independent growth-inhibitory and apoptosis-inducing activities of MLE in human cancer cells. 

Angiogenesis is a process of forming new blood vessels by endothelial cells from existing micro vessels (Hanahan and Weinberg, 2011[11]). Since the new blood grown into the primary tumor provide the necessary oxygen, nutrients, and growth factors for tumor progression, angiogenesis is regarded as an important target in cancer prevention and treatment (Granci et al., 2010[8]). Angiogenesis is triggered by the endogenous production of specific pro-angiogenic factors secreted from tumor cells (Folkman, 2002[6]). VEGF and bFGF are regarded as most potent mediators of angiogenesis (Folkman, 2002[6]; Gupta and Qin, 2003[10]), and VEGF appears to play a dormant role in different cancers, including cancers of colon and lung (Frandsen et al., 2016[7]). Our results indicate that MLE suppressed VEGF and bFGF secretion of HCT116 and H1299 cells (Figure 4(Fig. 4)). Particularly, the secretion of VEGF was almost nullified by the treatment with MLE (Figure 4(Fig. 4)). These results suggest inhibitory activities of MLE against angiogenic potentials of colon and lung cancer cells. Since VEGF and bFGF produced by primary tumor cells also contribute to promoting cell proliferation in an autocrine manner (Cao et al., 2012[3]; Berger et al., 1999[2]), the inhibition of pro-angiogenic factor secretion by MLE (Figure 4(Fig. 4)) may partially explain the growth-inhibitory activities of MLE (Figure 1(Fig. 1)).

Metastasis is a major cause of cancer-related death (Hanahan and Weinberg, 2011[11]), and therefore, extensive research has been conducted in order to identify effective anti-metastatic agents (Shu et al., 2010[30]). Invasion, migration, and adhesion to extracellular matrix are key events that neoplastic cells must undergo during metastatic cascade (Hanahan and Weinberg, 2011[11]). Our results indicate that MLE inhibited the invasion and adhesion in both HCT116 and H1299 cells as well as the migration in H1299 cells (Figures 5-7(Fig. 5)(Fig. 6)(Fig. 7)). To the best of our knowledge, this is the first study reporting such inhibitory activities of MLE as well as the inhibition of pro-angiogenic factor secretion (Figure 4(Fig. 4)) in human cancer cells. Such inhibitory activities of MLE was observed at the time points when cell viability was not affected (2-24 h) and, therefore, may not be due to the cytotoxicity of MLE.

The ethanol extract of mustard leaves used in the present study is a complex mixture containing different bioactive phytochemicals. The anticancer effects of cruciferous vegetables have been ascribed to glucosinolates and their hydrolysis products, including indoles and isothiocyanates (Higdon et al., 2007[12]). It needs to be further identified which constituent(s) is (are) most responsible for the inhibitory activities of MLE found in the present study. 

In summary, treatment with MLE dose-dependently inhibited the growth in HCT116 human colon cancer cells and H1299 human lung cancer cells, and this was accompanied by the induction of apoptosis. MLE was also effective in suppressing the anchorage-independent colony formation, pro-angiogenic factor secretion, invasion, and adhesion in HCT116 and H1299 cells as well as the migration in H1299 cells. Our results indicate that MLE possesses anti-cancer activities against colon and lung cancers *in vitro*. It still needs to be determined whether or not similar effects were reproduced in relevant animal models and finally humans. More studies are needed to demonstrate underlying cellular and molecular mechanisms of the inhibitory activities observed in the present study, which will help to better define the preventive and therapeutic potentials of MLE against human colon and lung cancers. 

## Conflict of interest

The authors declare that there is no conflict of interest.

## Acknowledgement

This work was supported by the National Research Foundation of Republic of Korea (No. 2010-0025311 & NRF-2015R1D1A1A 01059139).
